# The Evolution of Pesticide Resistance: A Data‐Driven Case Study of Chlorantraniliprole Resistance in *Chilo suppressalis* and Other Lepidopteran Pests in China

**DOI:** 10.1111/eva.70131

**Published:** 2025-07-06

**Authors:** Philip G. Madgwick, Russell Slater, Ricardo Kanitz

**Affiliations:** ^1^ Syngenta, Jealott's Hill International Research Centre UK; ^2^ Syngenta UK Cambridge UK; ^3^ Syngenta Crop Protection Basel Switzerland

**Keywords:** adaptation, contemporary evolution, diamide insecticides, evolutionary ecology, evolutionary genetics, parallel evolution, pesticide resistance management, resistance monitoring

## Abstract

Pesticide resistance presents some of the best examples of evolution by natural selection in action. An exceptionally well‐documented case from recent years is the evolution of resistance to the diamide chlorantraniliprole in the striped rice stem‐borer *Chilo suppressalis* in China. Prior to the registration of chlorantraniliprole, *C. suppressalis* had evolved resistance to almost all other available pesticides. Using data from resistance monitoring and laboratory analysis, the quantitative dynamics of chlorantraniliprole resistance evolution in *C. suppressalis* and other lepidopteran pests in China are collated and analysed. The results reveal the rapid evolution of high levels of chlorantraniliprole resistance in *C. suppressalis* causing control failure across China, primarily driven by the origin and spread of multiple identified major mutations of the target site. Some of the same mutations also drove the parallel evolution of chlorantraniliprole resistance in other lepidopteran pests. As well as providing an exceptional example of evolution by natural selection in action, the evolution of chlorantraniliprole resistance in *C. suppressalis* in China also provides a cautionary tale for resistance management.

## 1 Introduction

Pesticide resistance is often presented as providing some of the best examples of evolution by natural selection in action (Huxley 1953; Georghiou 1972; Forgash 1984; Metcalf 1989), though it has been neglected (Ford 1964; Endler 1986) or underrepresented (Kingsolver et al. 2001; Thurman and Barrett 2016) in influential academic surveys (though see (Reznick and Ghalambor 2001)). The commercial importance of pesticides has led to many cases of it being documented by long‐term, recurrent and widespread sampling (e.g., (Roditakis et al. 2018; Dandan et al. 2019; Domingues et al. 2019; Wang, Wang, et al. 2019; Zheng et al. 2021; İnak et al. 2022; Stavrakaki et al. 2023)). Consequently, there are numerous examples of pesticide resistance where the evolution of adaptation has been studied in extraordinary detail. An outstanding recent example with publicly available data and analysis is the evolution of resistance to the diamide chlorantraniliprole in the striped rice stem‐borer *Chilo suppressalis* in China.

*C. suppressalis* is a major pest of rice in Asia due to its devastating effects on crop yields (IRAC 2024b). Females can lay over 300 eggs on the underside of rice leaves. Larvae hatch to feed down the main vein of the leaf and into the stem. In rice seedlings, this can lead to ‘dead hearts’ where the borer kills the growing points of young shoots. In more mature plants, this can result in ‘white heads’ as empty grains are produced. A single larva can destroy multiple rice plants. Furthermore, its rapid lifecycle typically enables three to four generations per year in southern China (Meng et al. 2008). In the absence of control, *C. suppressalis* can cause > 95% yield loss (Vega and Heinrichs 1986). Consequently, chemical controls have been used for many years, but their use has been restricted by the evolution of resistance and the banning of some pesticides. From the early 1960s, organochlorides were used to control *C. suppressalis*, especially benzene hexachloride mixed with methyl parathion, which was later banned in China in 1983 (Su et al. 2014a). By this time, nereistoxins such as monosultap and organophosphates such as methamidophos had become popular, although methamidophos was also later banned in China in 2008. Monosultap and the organophosphate triazophos were popular throughout the 1990s, but by the end of the decade resistance had started to become a major problem (Qu et al. 2003; Cao et al. 2004). Fipronil started to become widely used from 1997, but resistance rapidly evolved to high levels by 2002, and it was later banned in China in 2009 (He et al. 2013; Su et al. 2014a). Because of a lack of alternatives, monosultap and triazophos continued to be used extensively into the late 2000s despite poor efficacy due to high levels of resistance. The new organophosphate chlorpyrifos also became popular because, remarkably for a pesticide with the same mode of action, it seemed to evade some of the resistance problems associated with triazophos. The new mode of action abamectin became available from 1998 and started to be used widely from 2006, but only in mixtures with triazophos or chlorpyrifos, which may partially explain why abamectin resistance evolved slowly (Su et al. 2014a; Meng et al. 2022).

When diamide insecticides became available for use against *C. suppressalis* in China from 2008, they provided a much‐needed new mode of action uncompromised by resistance. There are two types of diamide: anthranilic diamides such as chlorantraniliprole and phthalic diamides such as flubendiamide, both of which were registered for use against *C. suppressalis* in China in the same year. Anthranilic diamides have proved more successful; flubendiamide was later banned for use in rice in China in 2016, whilst other anthranilic diamides were developed and registered for use, including cyantraniliprole in 2012, tetrachlorantraniliprole in 2017 and tetraniliprole in 2020. Diamides are ryanodine receptor modulators, belonging in the insecticide mode of action classification group 28 (IRAC 2024a). Ryanodine receptors are large tetrameric channels found in the sarcoplasmic‐endoplasmic reticulum membrane in muscle and nerve tissue, controlling the release of calcium ions (Cordova et al. 2006; Sattelle et al. 2008). Diamides bind close to the C‐terminus to activate the receptor, causing feeding cessation, muscle contraction, general paralysis and death (Tonishi et al. 2005; Kato et al. 2009). The ryanodine receptor has a single isoform in insects with > 90% amino acid identity across lepidopteran pests; other insect orders have > 70% amino acid identity and mammals have three highly divergent isoforms (Cordova et al. 2006; Lahm et al. 2009; Wang et al. 2012). Consequently, anthranilic diamides such as chlorantraniliprole are broad‐spectrum insecticides with a desirable mammalian safety profile, well‐suited for the control of lepidopteran pests such as *C. suppressalis*. As such, chlorantraniliprole rapidly became intensively used after registration in 2008.

Here, information and data on the evolution of chlorantraniliprole resistance in *C. suppressalis* in China are collated to provide the first systematic documentation of what happened, with important implications for resistance management. A previous article (Richardson et al. 2020) has provided a scholarly review of the global status of diamide resistance across lepidopteran pests as a cross‐sectional study. Here, instead, the focus is on providing a longitudinal study through a data‐driven analysis of the quantitative dynamics of chlorantraniliprole resistance evolution in *C. suppressalis* in China from phenotypic and genotypic resistance monitoring. This analysis is supported by additional data from China on resistance to other pesticides in *C. suppressalis*, chlorantraniliprole resistance in other lepidopteran pests and resistance characterisation studies in the laboratory across lepidopteran pests.

## 2 Methods

Over many years, much work has been conducted into the resistance status of lepidopteran pests to chemical controls in China by academics with the support of industrial scientists at DuPont and Syngenta. Although many studies report relevant data, these have not been collated into datasets. To find relevant data, an informal literature search was undertaken manually following citation networks. This approach was taken for speed and because it led to the discovery of more relevant studies than other tested approaches (e.g., using specific search terms), especially because of language challenges (as some studies are only available in Chinese). All studies that reported data on resistance phenotypes, genotypes, mechanisms and properties were included in the analysis (as will be further described in turn); no studies were intentionally excluded. Besides *C. suppressalis*, the other lepidopteran pests of China with relevant data or information are *Cnaphalocrocis medinalis*, *Helicoverpa armigera*, *Plutella xylostella*, *Spodoptera exigua* , *Spodoptera frugiperda* and *Spodoptera litura* . All datasets are available on Dryad.

### 2.1 Resistance Monitoring of Quantitative Phenotypes in Field Populations

There are multiple methods of resistance monitoring, but a common method is to use a standardised bioassay to estimate the dose response curve for a sampled population (Miyata 1983; Roush and Miller 1986); see also (Finney 1947) for the underlying probit analysis. A key statistic is the lethal dose that kills 50% of the sampled individuals (LD50), which is used to calculate the resistance factor (or ratio; RF) as the multiplicative shift in the LD50 compared to a baseline measurement. The RF is a quantitative measurement of the mean level of resistance in a sampled population. It is important to note that it only quantifies the ‘benefits’ of resistance and not any of its pleiotropic costs. For *C. suppressalis*, most studies used the seedling dip method, where rice stems are dipped in a range of pesticide doses before contact with the pest (Gao et al. 2013). The study which provides the method for the standardised bioassay also describes the baseline LD50 (1.333 mg/larva), which is used as a reference for all RF calculations regardless of their location. Similar leaf or seedling dip bioassays and baselines were used for other lepidopteran pests in China (Cao et al. 2010; Wang et al. 2010, 2023; Lai et al. 2011; Zheng et al. 2011; Su et al. 2012). Minor differences between study methods were considered but ignored for the sake of RF calculation. Sample locations were reliably recorded as within a county, with only a few studies providing the longitude and latitude coordinates, and so the county designation was used for collating sample location.

### 2.2 Molecular Resistance Monitoring of Mutant Genotypes

Molecular resistance monitoring involves the identification of DNA markers that are associated with resistance mechanisms (Van Leeuwen et al. 2020). For chlorantraniliprole resistance in *C. suppressalis*, this is exclusively about estimating the frequency of known target‐site resistance mutations. To undertake cost‐effective molecular resistance monitoring, studies use microsatellite methods that only identify mutations that the array is designed to discover (rather than, say, DNA sequencing that could discover other mutations). Furthermore, as the number of individuals that are analysed (e.g., < 30) is often very small compared to population size, molecular resistance monitoring is only capable of detecting the frequency of common mutations (Denholm 1990); the detection of a mutation at zero frequency is interpreted with caution, as it may not reliably indicate mutation absence. Nonetheless, molecular resistance monitoring is a useful supplement to the resistance monitoring that quantifies the mean phenotype of field populations, allowing associations to be drawn between mutation presence and RFs.

### 2.3 Resistance Characterisation by Molecular and Genetic Analyses

The laboratory analysis of sampled populations can be used to characterise the nature and properties of resistance. Target‐site resistance mutations can be identified by DNA sequencing of the target site, which for chlorantraniliprole is the ryanodine receptor. Non‐target‐site resistance mutations are harder to identify. Metabolic resistance can be phenotypically detected by the overexpression or increased activity of known detoxification enzymes, including P450 monooxygenases, carboxylesterases, glutathione S‐transferases and ABC transporters. Across all characterisation studies of lepidopteran pests in China, the genotypic basis of metabolic resistance—such as other non‐target‐site resistance mechanisms—is never identified. The dominance of resistance can be quantified by crossing individuals from a sampled field population with a susceptible laboratory population. Note that this meaning of dominance here (from (Stone 1968)) is the distance that the LD50 of the resulting F1 offspring population is between the LD50 of the parent strains on the scale between −1 and +1 and is not a direct measure of the functional (or effective) dominance in terms of the relative mortality at a given dose. The fitness costs of resistance can also be quantified by measuring the life history properties of the different populations (Kliot and Ghanim 2012). Such analyses restrictively calculate the net fitness cost of resistance as a decrease in reproductive output under laboratory conditions (that are likely to be very different from field conditions).

### 2.4 Experimental Evolution of Resistance in Laboratory Populations

By either applying or withdrawing artificial selection from a pest in the laboratory, experimental evolution can be used to analyse the scope of resistance evolution in field populations. By applying chlorantraniliprole selection to a susceptible laboratory population, resistance can be evolved. As a means of forward genetics, the characterisation of resistance in evolved populations can then be used to suggest how resistance might evolve in field populations (McKenzie and Batterham 1998), assuming that the same resistance mechanism would drive resistance in both settings. The phenotypic response to a known strength of selection can be used to estimate the narrow‐sense heritability of the evolved mechanism of resistance (Tabashnik 1992). Alternatively, by withdrawing chlorantraniliprole selection from a field population in the laboratory, the net fitness cost of resistance can be qualitatively demonstrated.

## 3 Results

After searching through scientific publications, large datasets on *C. suppressalis* and other lepidopteran pests in China from field populations at widespread locations over many years were collated. There were 4602 resistance monitoring datapoints from 1996 to 2022 across seven lepidopteran pests, of which 328 related to chlorantraniliprole resistance in *C. suppressalis* (Table 1). Whilst there are datapoints from 15 provinces, > 80% of datapoints come from six provinces (in order of most datapoints to least): Zhejiang, Hubei, Jiangsu, Jiangxi, Hunan and Anhui. There were 140 molecular resistance monitoring datapoints from 2011 to 2020 for chlorantraniliprole resistance across four lepidopteran pests, of which 53 related to *C. suppressalis*. Whilst there are datapoints from eight provinces, > 80% of datapoints come from three provinces (in order): Zhejiang, Jiangxi and Hunan. There were 12 chlorantraniliprole resistance characterisation estimates across four lepidopteran pests: three for dominance, three for fitness cost and six for narrow‐sense heritability. Finally, there were 364 laboratory datapoints for up to 52 generations of experimental evolution across four lepidopteran pests, of which 179 related to chlorantraniliprole resistance.

**TABLE 1:** Resistance phenotype (i.e., RF) datapoints for lepidopteran pests in China.

Pest	Major host crop(s)	First year	Last year	Chlorantraniliprole datapoints	Total datapoints
*Chilo suppressalis*	Rice	2000	2022	328	1570
*Cnaphalocrocis medinalis*	Rice	2005	2022	59	432
*Helicoverpa armigera*	Cotton, corn, vegetables	1996	2019	22	117
*Plutella xylostella*	Cruciferous vegetables	1999	2018	90	857
*Spodoptera exigua*	Cotton, corn, vegetables	1998	2019	84	656
*Spodoptera frugiperda*	Corn, soya bean, vegetables	2019	2022	83	442
*Spodoptera litura*	Cotton, soya bean, vegetables	2004	2022	51	528

### 3.1 Resistance Monitoring of Quantitative Phenotypes in Field Populations

**FIGURE 1:**
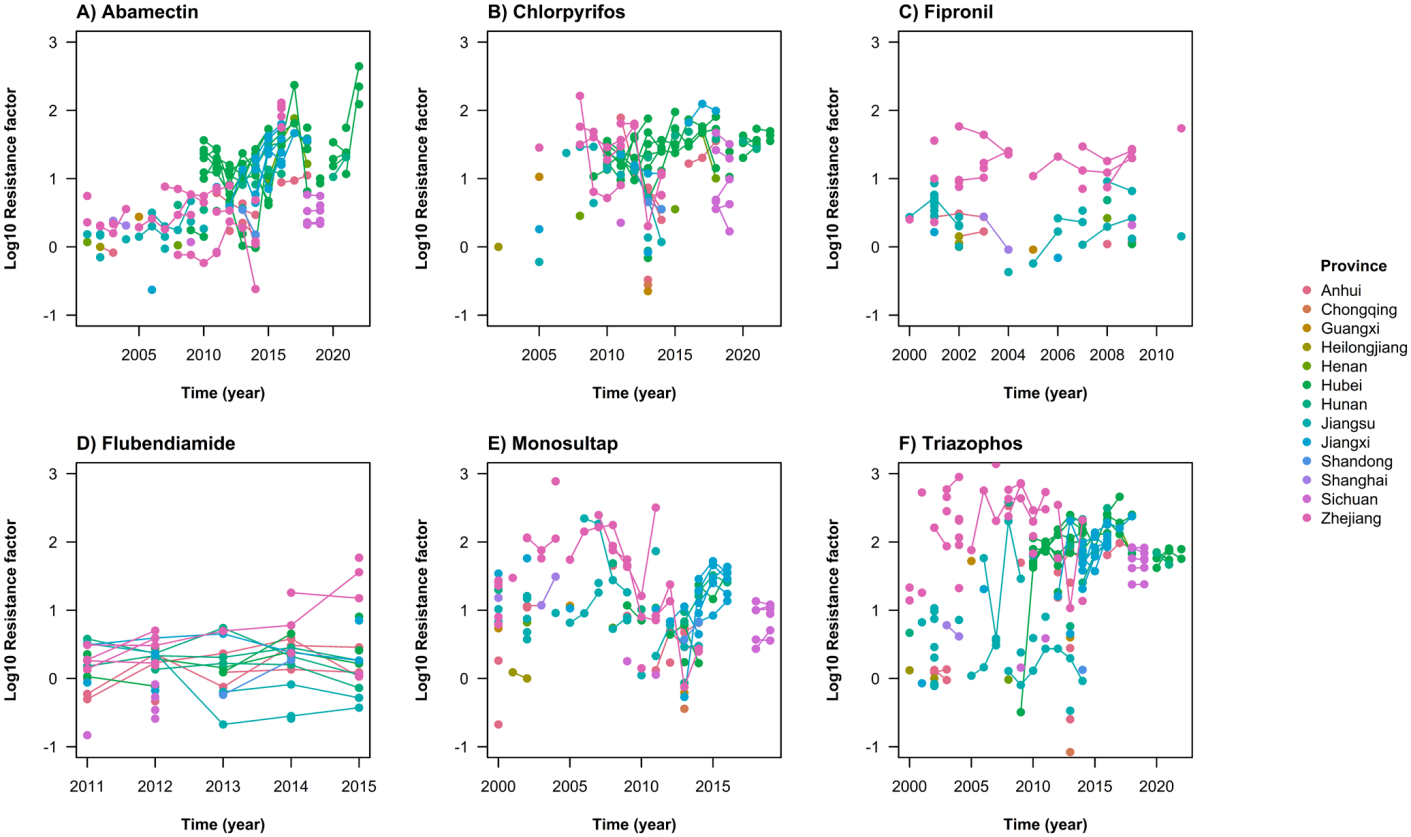


A large number of studies reported RF data for *C. suppressalis* in China (Cao et al. 2001, 2004; Han et al. 2003; Qu et al. 2003; Cao and Shen 2005; Huang et al. 2005, 2020; Huang, Sun, et al. 2021; Jiang et al. 2005; He, Chen, et al. 2007; He, Gao, et al. 2007; He et al. 2008, 2012, 2014; Chen et al. 2009; Cheng et al. 2010; Hu et al. 2010; Gao et al. 2013; Su et al. 2014b, 2023; Wu et al. 2014; Zhang, Wang, et al. 2014; Lu et al. 2017; Shuijin et al. 2017; Yao et al. 2017; Zhao et al. 2017, 2021; Sun et al. 2018; Sun, Wang, et al. 2023; Mao et al. 2019; Wei et al. 2019; Meng et al. 2022; Ma et al. 2023). Data for resistance to pesticides other than chlorantraniliprole confirm the expectations of high RFs in *C. suppressalis* to the available pesticides prior to diamide registration in 2008 (Figure 1). Monosultap, triazophos and chlorpyrifos resistance is consistently at high RFs over the sampling timeframe. Fipronil and flubendiamide resistance were at low RFs up until these modes of action were banned. Abamectin shows a slow, exponential (i.e., log‐linear) increase in the RF.

**FIGURE 2:**
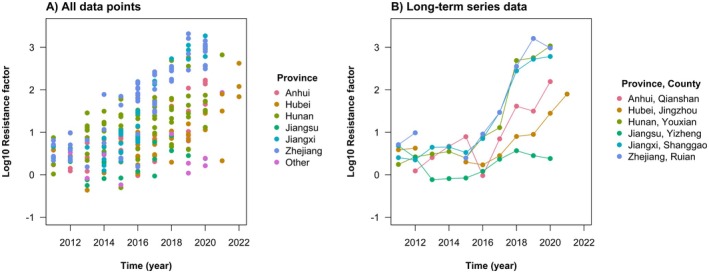


**FIGURE 3:**
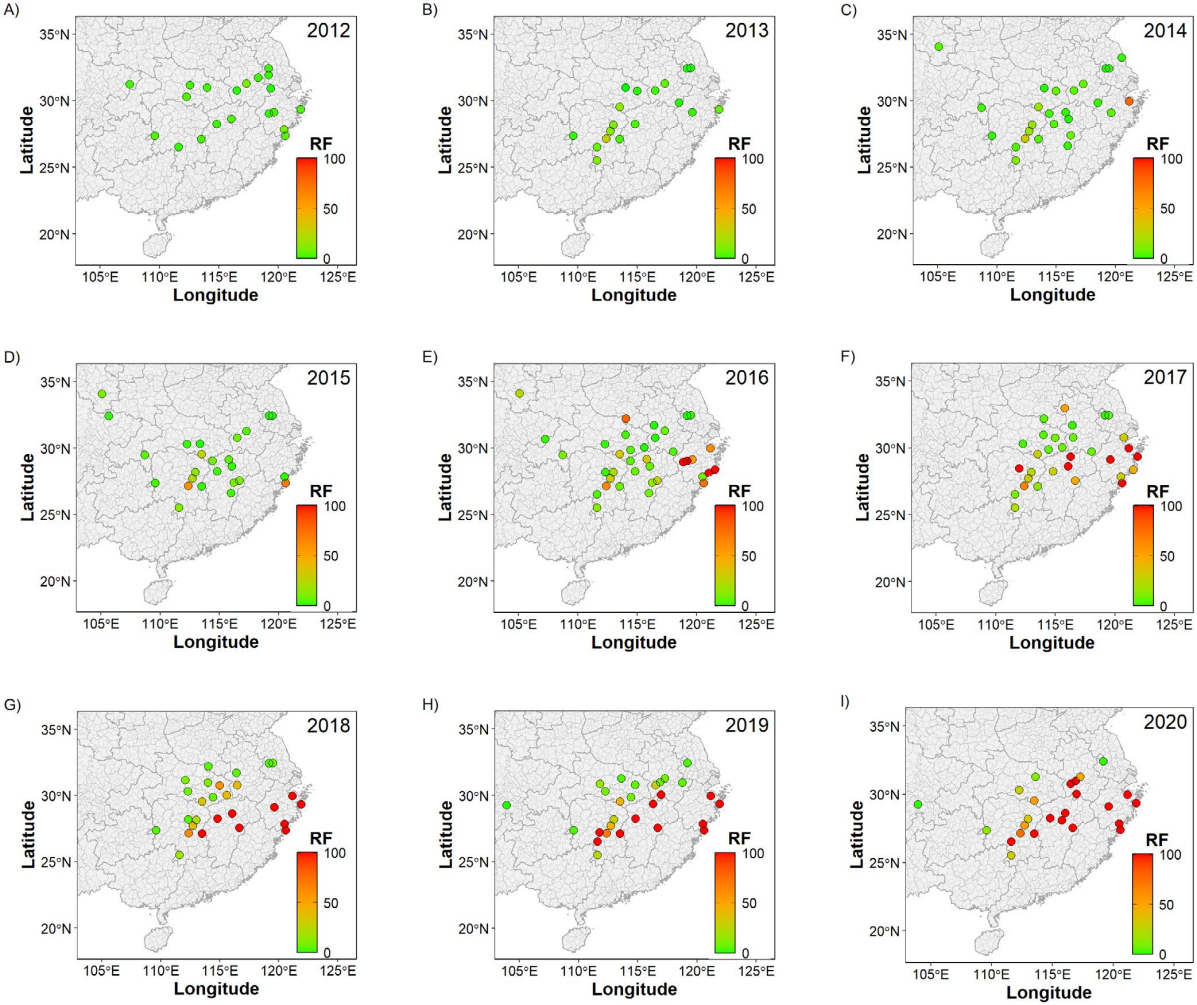


Data for chlorantraniliprole resistance show a clear general pattern of increasing RF values across locations over time (Figure 2A). In locations where the RF was increasing, the rate of increase tended to be exponential but was punctuated by short periods with an accelerated rate of increase. This pattern can be most clearly seen when extracting the locations that had the longest time series data for each key province (Figure 2B). Plotting the RF datapoints onto a time series of maps of China shows the apparent spread of high levels of resistance from a few locations (Figure 3). This is consistent with the estimated mobility of *C. suppressalis*, which commonly disperses < 3 km per generation but is thought to be able to disperse up to 20 km (Sun et al. 1993); see also supporting genetic analysis (Meng et al. 2008; Liu et al. 2013; Tang et al. 2016). Before 2012, low RF values were recorded across China. In 2013, resistance was reported to IRAC in the inland county of Gongan in Hubei province (IRAC 2014). The following year in 2014, a high RF was reported in the coastal county of Yuyao in Zhejiang province (Yao et al. 2017)—over 850 km away. After that, there is a period of spatial heterogeneity in the recorded RFs, with high RFs at some locations—especially at those close to Gongan and Yuyao counties—and low RFs at others—including some near Gongan and Yuyao counties. Field efficacy was reported to have more gradually declined in these regions (Richardson et al. 2020), with reports of control failure associated with the measurement of RFs over 50. From 2017 onwards, increasingly consistent high RFs over 100 have been recorded in many locations, as resistance starts to become widespread throughout the major rice growing regions in central China.

**FIGURE 4:**
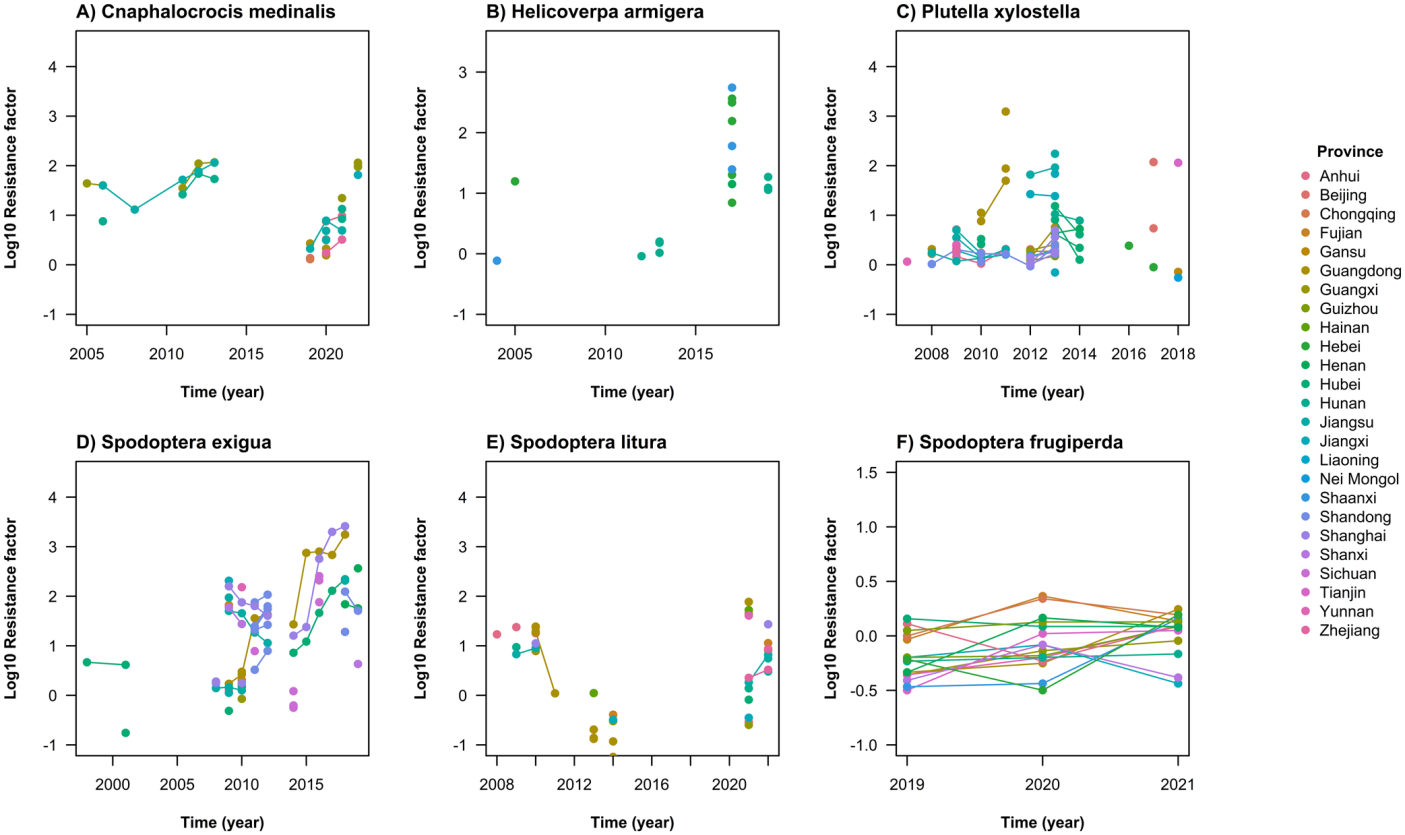


Other studies have reported RF data for other lepidopteran pests in China: *C. medinalis* (Zheng et al. 2011; Zhang, Ren, et al. 2014; Sun, Liu, et al. 2023), *H. armigera* (Cao et al. 2010; Zhang et al. 2018; Tang et al. 2021; Wang, Rui, et al. 2021), *P. xylostella* (Wang et al. 2010; Wang, Wang, et al. 2019; Wang, Zheng, et al. 2021; Zhou, Huang, and Xu 2011; Wang and Wu 2012; Gong et al. 2013; Shao et al. 2013; Xia et al. 2014; Jiang et al. 2015; Li et al. 2015; Hu et al. 2016; Zhang et al. 2016; Yin et al. 2019), *S. exigua* (Jia and Shen 2008; Si et al. 2009; Lai et al. 2011; Zhou, Liu, et al. 2011; Che et al. 2013; Su and Sun 2014; Zhang, Gao, et al. 2014; Wang, Xiang, et al. 2018; Wang, Yang, et al. 2021; Huang, Zhao, et al. 2021), *S. frugiperda* (Zhao et al. 2020; Lv et al. 2021; Wang et al. 2022, 2023; Zhu et al. 2023) and *S. litura* (Huang and Han 2007; Su et al. 2012; Tong et al. 2013; Pan et al. 2014; Sang et al. 2016; Wang, Huang, et al. 2018; Wang, Lou, and Su 2019; Zhang et al. 2022; Che et al. 2023). The RF datapoints relating to chlorantraniliprole (Figure 4) exhibit some common problems with RF datasets in the literature, with inconsistent sampling over time and space, reflecting how the sampling is undertaken to establish a baseline and then confirm a reported case of resistance. Nonetheless, a similar general pattern to *C. suppressalis* of increasing RF values across locations over time is discernible. There are obvious cases where there are accelerated rates of RF increase, especially for *P. xylostella* and *S. exigua* .

### 3.2 Molecular Resistance Monitoring of Mutant Genotypes

**FIGURE 5:**
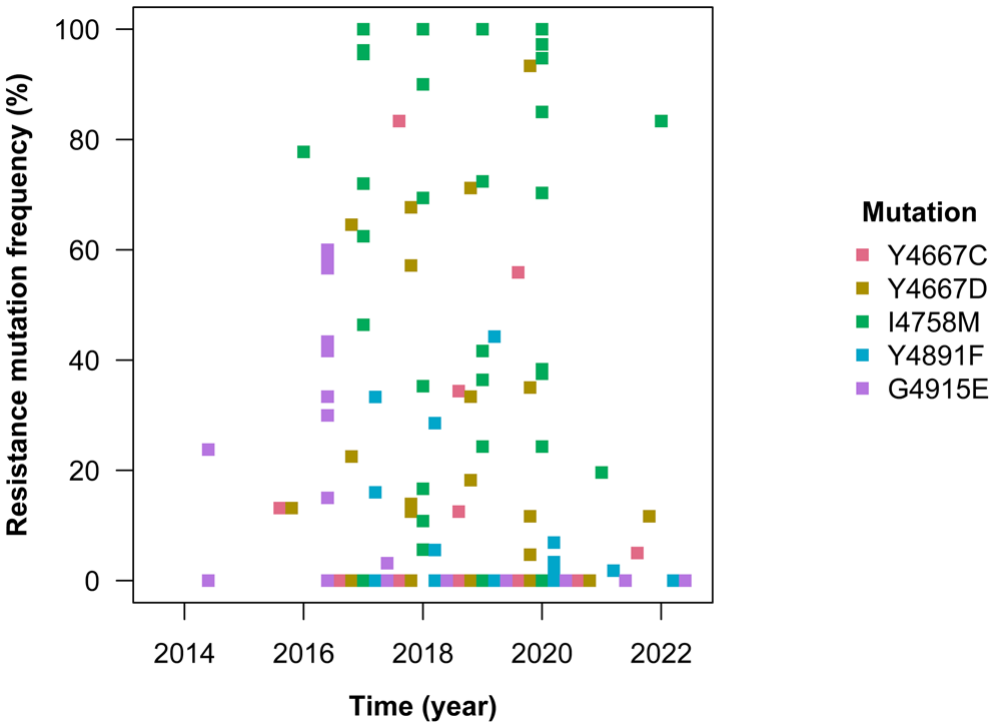


Some studies reported mutation frequency data for *C. suppressalis* in China (Lu et al. 2017; Yao et al. 2017; Sun et al. 2018; Sun, Wang, et al. 2023; Wei et al. 2019; Huang et al. 2020; Huang, Zhao, et al. 2021). The mutations relate to five target‐site mutations in the ryanodine receptor identified in *C. suppressalis* in China that confer resistance to chlorantraniliprole (Figure 5), which have been strongly implicated in causing high RFs by genome editing (Huang et al. 2020; Huang, Sun, et al. 2021). In this way, molecular resistance monitoring has identified a role for multiple mutations and mutational steps. To describe what happened, the early reports of resistance in 2013 and 2014 (IRAC 2014; Yao et al. 2017) must be distinguished because they were driven by independent mutation events.

In the coastal regions near Yuyao county, the G4915E mutation rapidly spread to high frequency between first detection in 2014 and near fixation in 2016 in association with high RFs over 100 (Lu et al. 2017; Yao et al. 2017). RF data from transgenic *Drosophila melanogaster* with the *C. suppressalis* ryanodine receptor suggest that, whilst single mutants with this one mutation have a high RF over 100, double mutants with this mutation and any other identified mutation tend to have a lower RF (Huang et al. 2020; Huang, Sun, et al. 2021), indicating that it may be an evolutionary dead‐end in *C. suppressalis*. The G4915E mutation was then rapidly replaced by single mutants with the I4578M mutation, which was found at near fixation in many populations from 2017 in association with RFs as high as 355 (Sun et al. 2018; Sun, Wang, et al. 2023; Wei et al. 2019; Huang et al. 2020; Huang, Sun, et al. 2021). Puzzlingly, RF data from transgenic *D. melanogaster* suggest that single mutants with the I4758M mutation would have a lower RF than single mutants with the G4915E mutation (Huang et al. 2020; Huang, Sun, et al. 2021). From 2019, double mutants with the I4578M and Y4667C mutations have been detected across coastal regions including Yuyao county in association with high RFs over 1000 (Huang, Sun, et al. 2021).

In inland regions near Gongan county, the G4915E mutation has rarely been detected (Lu et al. 2017). Instead, the I4758M mutation rapidly spread to high frequency between the first detection in 2013 and near fixation in 2017 in association with RFs over 100 (Sun et al. 2018; Sun, Wang, et al. 2023; Wei et al. 2019; Huang et al. 2020; Huang, Sun, et al. 2021). From 2017, the Y4667D mutation has seemingly rapidly replaced the I4578M mutation in some sampled populations, such as Nancheng county in Jiangxi province, driving a shift towards RFs over 1000 (Huang et al. 2020; Huang, Sun, et al. 2021). Whilst the replacement is consistent with RF data from transgenic *D. melanogaster* (Huang et al. 2020; Huang, Sun, et al. 2021), the mutation does not appear to have become widespread in inland regions, suggesting that other factors might be at play. From 2019, double mutants with the Y4667D and Y4891F mutations have been detected in association with high RFs around 1000 but only in Nancheng county (Huang, Sun, et al. 2021).

**FIGURE 6:**
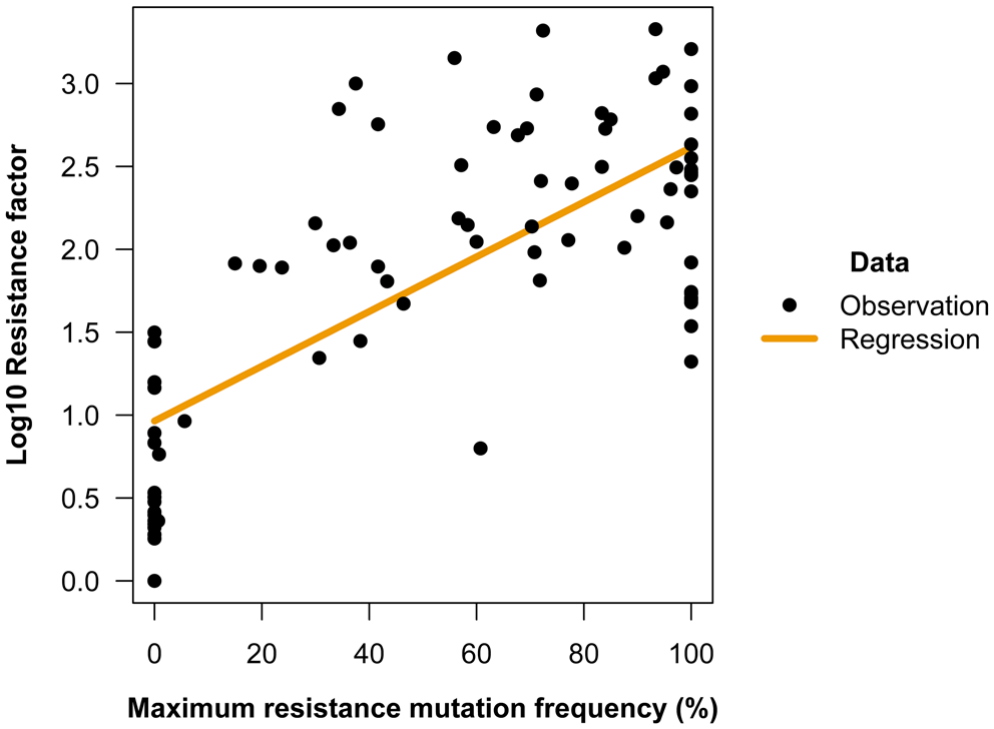


Other studies have reported mutation frequency data for other lepidopteran pests in China: *C. medinalis* (Sun, Liu, et al. 2023), *P. xylostella* (Guo, Liang, et al. 2014; Guo, Wang, et al. 2014; Steinbach et al. 2015; Shen et al. 2023), *S. exigua* (Zuo et al. 2020; Huang, Zhao, et al. 2021) and *S. frugiperda* (Lv et al. 2021). Similar target‐site resistance mutations to those found in *C. suppressalis* have been identified across lepidopteran pests. In the absence of genome editing data to confirm the sufficiency of target‐site resistance mutations causing high RFs (though see (Zuo et al. 2017) for support for one mutation), the high frequency of at least one identified target‐site resistance mutation can be shown to be correlated with high RFs across the lepidopteran pests (Figure 6; linear regression adj‐*R* ^2^ = 0.5239). This analysis is simple but imperfect; there is no reason to think that different populations or pests would have the same logarithm of RFs when mutations have 0% frequency because of natural variation in their resistance baselines or, in addition to such background effects, when they have 100% frequency because the mutation with maximum frequency can be different—or even, regardless, that there would be a linear relationship between mutation frequency and the logarithm of RFs. With this in mind, the correlation is surprisingly strong, providing critical evidence of the association between target‐site mutation presence and high RFs in the absence of other available evidence.

Orthologous mutations to those in *C. suppressalis* have been found in other lepidopteran pests. For *P. xylostella* in China, five target‐site resistance mutations have been identified (G4946E, I4790K, I4790M, E1338D and Q4594L; (Guo, Liang, et al. 2014)), of which two have orthologs in *C. suppressalis* (G4915E, I4758M). Two mutations (G4946E and E1338D) appear to have become common and widespread as a double mutant (Shen et al. 2023). Despite *S. frugiperda* having only been an invasive pest in China since 2018 and having remained broadly susceptible to chlorantraniliprole (whilst having evolved resistance to other pesticides), familiar target‐site resistance mutations have still been found (Sun et al. 2021; Wang et al. 2023; Zhu et al. 2023). Two (likely single) mutations have been identified at low frequencies (G4891V and I4743L; (Lv et al. 2021)), which are orthologous to G4915E and I4758M in *C. suppressalis*. Only one target‐site resistance mutation has been detected in *C. medinalis*, *S. exigua* and *S. litura* in China: I4712M (Sun, Liu, et al. 2023), I4743M (Zuo et al. 2020; Huang, Zhao, et al. 2021) and I4728M (Mei et al. 2023) respectively, which are all orthologous to I4758M in *C. suppressalis*. In *S. exigua* , genome editing has suggested that an ortholog of the G4915E could cause high levels of resistance, but it has not been found in the field (Zuo et al. 2017). Finally, it is worth stating that *H. armigera* in China remains broadly susceptible to chlorantraniliprole (Wang, Rui, et al. 2021), having been successfully controlled through *Bt* crops for over 20 years (Dandan et al. 2019). Higher RFs have been recorded in recent years (Figure 4B), but no target‐site resistance mutations have yet been implicated.

### 3.3 Resistance Characterisation by Molecular and Genetic Analyses

The association between high RFs and target‐site resistance mutations from phenotypic and genotypic resistance monitoring provides strong evidence for the role of target‐site mutations in chlorantraniliprole resistance. Furthermore, for *C. suppressalis*, transgenic *D. melanogaster* have been used to confirm the sufficiency of the identified target‐site resistance mutations in causing a high RF (Huang et al. 2020; Huang, Sun, et al. 2021). The role of non‐target‐site resistance mechanisms is more difficult to ascertain. Metabolic resistance has been suggested to play a role in chlorantraniliprole resistance in *C. suppressalis* in China through the upregulation of carboxylesterases (Lu et al. 2017), but the evidence is weak (Yao et al. 2017). The situation is similar across the lepidopteran pests of China (Liu et al. 2015; Troczka et al. 2017; Zuo et al. 2020; Lv et al. 2021; Wang, Rui, et al. 2021; Che et al. 2023; Sun, Liu, et al. 2023; Sun, Lu, et al. 2023). The most convincing evidence of the role of non‐target‐site resistance mechanisms across the lepidopteran pests in field populations to date comes from those sampled populations where no target‐site resistance mutations have been detected, which can have RFs that have increased to as high as 32 in *C. suppressalis* (Huang, Sun, et al. 2021).

Crosses between field and laboratory populations with known genotypes can be used to estimate the dominance of chlorantraniliprole resistance, which is particularly relevant when field populations are known to harbour target‐site resistance mutations (Table 2). There are no dominance estimates for chlorantraniliprole resistance in *C. suppressalis*, but the estimates from other lepidopteran pests in China are −0.89 for a laboratory population of *S. exigua* that has been genome‐edited to have the ortholog of the G4915E mutation and a RF of 223 (Zuo et al. 2017), −0.95 for a field population of *S. exigua* with the ortholog of the I4758M mutation and a RF of 21 (Zuo et al. 2020) and −0.22 for a field population of *P. xylostella* that very likely had the ortholog of the G4915E mutation and a RF of 2040 (Wang et al. 2013). As such, the key target‐site resistance mutations appear to be incompletely recessive to varying extents.

**TABLE 2:** Estimates of the dominance, fitness cost and narrow‐sense heritability of chlorantraniliprole resistance from resistance characterisation and experimental evolution.

Pest	Parameter	Generations	Mortality	RF	Estimate	Reference(s)
*Plutella xylostella*	Dominance	—	—	2040	−0.22	(Wang et al. 2013)
*Spodoptera exigua*	Dominance	—	—	223	−0.89	(Zuo et al. 2017)
*Spodoptera exigua*	Dominance	—	—	21	−0.95	(Zuo et al. 2020)
*Chilo suppressalis*	Fitness cost*	—	0%	10.6	0.53	(Sun, Wang, et al. 2023)
*Plutella xylostella*	Fitness cost	—	0%	52.5	0.55	(Gong et al. 2014)
*Spodoptera exigua*	Fitness cost	—	0%	12	0.25	(Liu et al. 2021)
*Chilo suppressalis*	Heritability*	10	30%	10.6	0.387	(Sun, Wang, et al. 2023)
*Choristoneura rosaceana*	Heritability	11	70%	8.5	0.151**	(Sial and Brunner 2012)
*Plutella xylostella*	Heritability	18	30%–50%	52.5	0.26	(Gong et al. 2014)
*Plutella xylostella*	Heritability	52	70%	48.17	0.069**	(Liu et al. 2015)
*Spodoptera exigua*	Heritability	22	70%	12.95	0.1082	(Lai and Su 2011)
*Spodoptera exigua*	Heritability	6	30%	227	0.058	(Liu et al. 2021)

* Selection from tetraniliprole not chlorantraniliprole.

** Calculated from data following the method in (Tabashnik 1992).

The fitness cost of chlorantraniliprole resistance can be quantified by measuring the life history properties of the different populations (Table 2). There are no fitness cost estimates for chlorantraniliprole resistance in *C. suppressalis*, but there is an estimate of a laboratory population of *C. suppressalis* that evolved under tetraniliprole selection to a RF of 10.6, having a relative fitness of 0.53 compared to the original laboratory population (Sun, Wang, et al. 2023). The fitness cost is likely associated with non‐target‐site resistance mechanisms. There are fitness cost estimates for chlorantraniliprole resistance in other lepidopteran pests, which again are calculated for laboratory populations evolved under artificial selection and likely associated with non‐target‐site resistance mechanisms: a relative fitness of 0.55 for *P. xylostella* with a RF of 52.5 (Gong et al. 2014) and 0.25 for *S. exigua* with a RF of 12 (Liu et al. 2021).

### 3.4 Experimental Evolution of Resistance in Laboratory Populations

**FIGURE 7:**
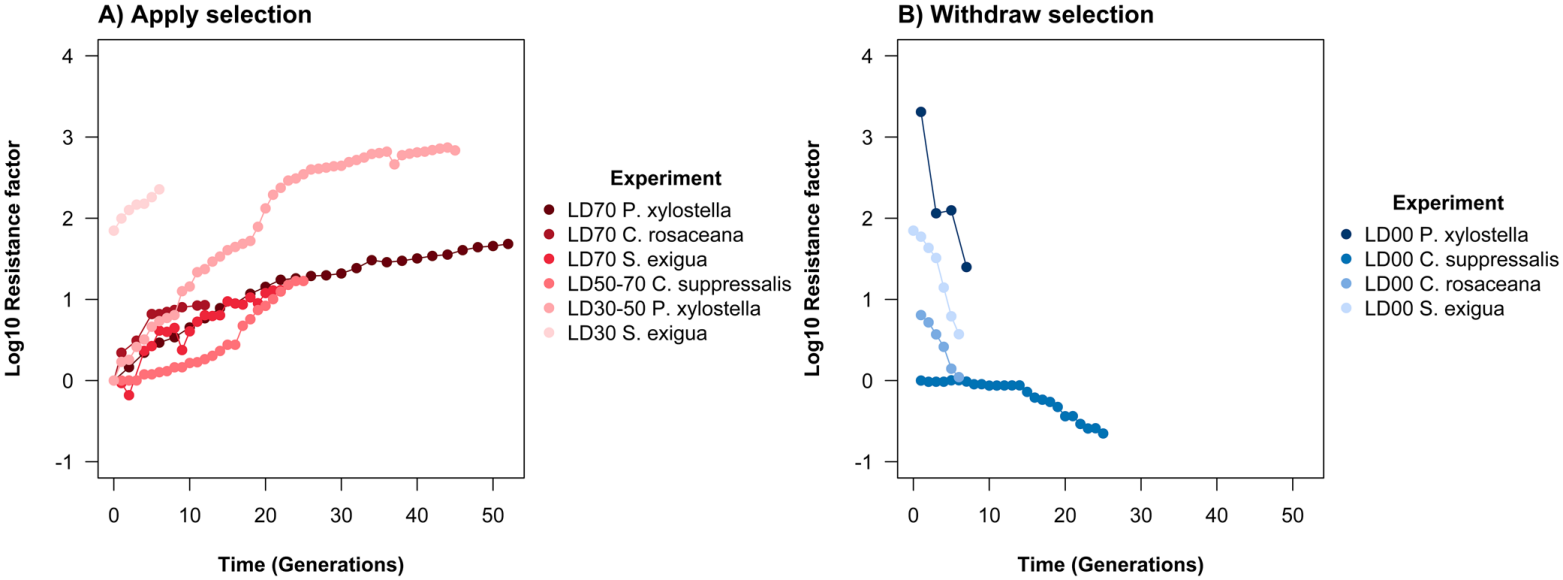


Experimental evolution can impose continuous artificial selection on a laboratory population using chlorantraniliprole at a specified lethal dose. High RFs can be produced over a small number of generations (Figure 7A). No evidence of target‐site resistance mutations has ever been found in these studies (Qin et al. 2018), but there is clear evidence of metabolic resistance through the upregulation of detoxification enzymes including P450 monooxygenases and ABC transporters (Xu et al. 2019; Peng et al. 2021). Experimental evolution can be used to estimate the narrow‐sense heritability of the non‐target‐site resistance mechanism(s). There are no estimates of the heritability of chlorantraniliprole resistance for *C. suppressalis*, but there is an estimate after 10 generations of tetraniliprole selection at LD30 of 0.387 (Sun, Wang, et al. 2023). Chlorantraniliprole selection in other lepidopteran pests yields slightly lower heritability estimates in the range between 0.058 for *S. exigua* after 6 generations (Liu et al. 2021) and 0.26 for *P. xylostella* after 18 generations (Gong et al. 2014).

Alternatively, experimental evolution can withdraw selection on a laboratory or field population, imposing no mortality from chlorantraniliprole. Field populations with high RFs can see drastic reductions in the RF over time (Figure 7B), especially if there is a target‐site resistance mechanism (Wang et al. 2013; Liu et al. 2021), providing strong evidence of the high fitness costs of target‐site resistance. There are no experiments using resistant field populations of *C. suppressalis*. For a field population of *P. xylostella* with a RF of 2040 that very likely had the ortholog of the G4915E mutation, the RF declined to 25 in 7 generations (Wang et al. 2013) and, for a field population of *S. exigua* with a RF of 70, the RF declined to 4 within 6 generations (Liu et al. 2021). Equally, non‐target‐site resistance mechanisms also appear costly, as a laboratory‐evolved population of *Choristoneura rosaceana* with a RF of 6.4 declined to 1.1 in 6 generations (Sial and Brunner 2012); note that this is not a major pest in China, but it is another related lepidopteran pest. It is also noteworthy that susceptible field populations can see reductions in the RF over time, as shown for a population of *C. suppressalis* with a RF of 1 that declined to 0.22 in 25 generations (Xu et al. 2019), which is likely to be due to laboratory adaptation.

## 4 Discussion

Pesticide resistance can provide some of the best examples of evolution by natural selection in action, as demonstrated by the extraordinarily well‐documented case of chlorantraniliprole resistance in populations of *C. suppressalis* across China. Other celebrated examples of the long‐term evolution of adaptation tend to either involve documenting the effects of uncontrolled selection pressures in small, wild populations of charismatic organisms (e.g., beak evolution in Darwin's finches (Grant and Grant 2014)) or mapping out evolutionary trajectories in response to simple, artificial selection in microorganisms using replicates (e.g., long‐term evolution experiments in *E. coli* (Good et al. 2017)). Semi‐natural conditions have long been recognised as bringing together some of the most desirable features of field and laboratory studies, such as in the case of industrial melanism in the peppered moth *Biston betularia* (Majerus 1998). The semi‐natural conditions of pesticide resistance evolution are an exceptional case, where a novel selection pressure is introduced by a single, abrupt change in the environment to a large, wild population of complex, multicellular organisms. Whilst technical replicates may not be possible, the application of the same selection pressure in different locations and to closely related pests provides a kind of replication. Furthermore, the commercial importance of pesticides has driven the long‐term, recurrent and widespread sampling of resistance phenotypes and genotypes—alongside resistance characterisation and experimental evolution in the laboratory—affording an extraordinary depth and breadth of understanding, as is clearly demonstrated here.

### 4.1 Summary of the Case Study

The evolution of chlorantraniliprole resistance in *C. suppressalis* in China was rapid. Prior to chlorantraniliprole registration in 2008, *C. suppressalis* had evolved high resistance factors (RFs; i.e., fold increases in the lethal dose that kills 50% of individuals in a standardised bioassay) to the available pesticides (Figure 2). From chlorantraniliprole registration in 2008 to the first reports of resistance in 2013 and 2014, there was little (or no) increase in the RF (Figure 3); differences in the measured level of resistance reflect natural and fluctuating variation in the susceptibility baseline across China. The first reports of resistance—which were associated with reports of control failure from farmers—involved the detection of high RFs around 100 in locations where low RFs around 1 had been recorded in the year(s) before. This abrupt evolutionary dynamic is as expected when a rare mutation of major effect exponentially increases in frequency to become common (Haldane 1924).

Genetic analysis went on to confirm the high frequency of a target‐site resistance mutations (G4915E or I4758M), with different mutations becoming common in coastal and inland locations. Over time, one mutation (I4758M) became common in both regions. The same single nucleotide polymorphism is responsible for the amino acid change in both locations, which might suggest that this substitution was driven by migration; but many populations in between these regions only latterly acquired the mutation, which is more suggestive of the localised spread of the same mutation from independent mutation events. This is further supported by the geographic barrier of the Nanling and Wuyi mountains that separate the coastal and inland provinces. From 2016 to 2018, high RFs are recorded at increasingly distant locations from the first reports of resistance (Figure 4). The invasion dynamic is as expected following a wave of advance (Fisher 1937).

The subsequent evolution of resistance was more complicated. From 2016, metabolic resistance was arguably suggested to have been detected; regardless, rare populations without target‐site resistance mutations were recorded with RFs over 10. The combination of target‐site and non‐target‐site resistance mechanisms may explain the evolution of RFs over 1000. From 2019, resistance was widespread, with high RFs being recorded throughout China. In some populations, double target‐site resistance mutations were detected (I4578M and Y4667C, or Y4667D and Y4891F). The detection of one of those double mutations (I4578M and Y4667C) at multiple locations suggests that it may be spreading into the local area around its location of origin.

### 4.2 Parallel Evolution in Other Lepidopteran Pests

There are some particularly striking features of the genetics of adaptation in this case study, which require some further elucidation. In the large population of *C. suppressalis* in China, five single‐step, target‐site resistance mutations have been detected at high frequencies over time at different locations (Figure 5), but only one has approached fixation across China (I4578M). It seems likely that allelic competition between these mutations that are physical neighbours on a chromosome has led to the near fixation of the most adaptive. Genome editing suggested that a different mutation had a higher RF (G4915E), but of course the RF is only an assessment of the mutation's benefits—and the selection coefficient depends on its net fitness benefits *and costs*; the existence of mutation costs can be demonstrated by the decline in the RF after withdrawing chlorantraniliprole selection (Figure 7B).

Orthologous mutations have been found at high frequency across four other lepidopteran pests, underlying the evolution of high RFs in China, albeit that a mutation with a different ortholog (G4915E) appears to be the most adaptive in *P. xylostella*. Outside of China (i.e., through independent evolutionary events), these same two mutations (G4915E or I4758M) have been found at high frequencies across lepidopteran pests (Steinbach et al. 2015; Roditakis et al. 2017; Boaventura, Bolzan, et al. 2020; Boaventura, Martin, et al. 2020; Shen et al. 2023). Presumably, in lepidopteran pests where chlorantraniliprole resistance has not yet been characterised, these mutations are also likely to play an important role (Uchiyama and Ozawa 2014; Hafez et al. 2019; Contini et al. 2022), including for *H. armigera* in China.

With the early stages of the evolution of double target‐site resistance mutations ongoing, it is unclear whether this pattern of near‐fixing the most adaptive mutations will continue. Interestingly, the spreading double target‐site resistance mutations (I4578M and Y4667C) are now two mutational steps away from the predicted optimum (I4758M and Y4667D) from genome editing (Huang et al. 2020; Huang, Sun, et al. 2021). Perhaps, as the possible mutational trajectories proliferate, the global fitness optimum may be less likely to occur—even with the large population size.

### 4.3 Limited Value of Some Laboratory Analyses

Some laboratory analyses were of great value to the understanding of chlorantraniliprole resistance evolution in *C. suppressalis* in China, but they also had their limitations. There was enormous value to establishing the resistance mechanisms at work in field populations, which underlie the molecular resistance monitoring data. Without this analysis, it would not have been possible to link the spread of different levels of resistance to their underlying causes. Yet, far too little attention was paid to quantifying the contributions of different resistance mechanisms and mutations. No study has ever identified a metabolic resistance mutation. Only one study has tried to quantify the contribution of metabolic resistance to the overall RF; piperonyl butoxide (PBO), diethyl maleate (DEM) and triphenyl phosphate (TPP) were used to suppress metabolic resistance mechanisms in *S. litura* , resulting in a drop of the RF from 77 to 28, 33 and 71 respectively (Che et al. 2023), suggesting that metabolic resistance accounted for up to 2.7 of the RF (or 6.9 if each synergist were treated as acting independently, which is not implausible whilst the metabolic resistance mechanism remains unknown).

Other laboratory analyses were of much more limited value to understanding field results, such as those estimating the dominance, fitness costs and narrow‐sense heritability, but still found some surprising findings. Dominance (in the sense of (Stone 1968)) estimates are difficult to translate into an estimate of the functional (or effective) dominance that governs the rate of heterozygote selection in the field because of the controlled exposure of the pest to the pesticide under laboratory conditions. Yet, whilst studies that measured dominance failed to directly test how it relates to identified resistance mechanisms, surprisingly, it was possible to infer the presence of target‐site mutations in all cases from other studies, suggesting that the mutations were incompletely recessive up to −0.95 (Table 2).

Fitness costs were also only quantified under laboratory conditions and, more importantly, were only estimated for laboratory‐evolved populations with metabolic resistance, which does not reflect the target‐site resistance of field populations. Nonetheless, the estimates of the fitness costs of metabolic resistance were surprisingly high, including up to a 75% decrease in relative fitness (Table 2), which is unexpected for non‐target‐site resistance mechanisms (Lande 1983; Macnair 1991; Groeters and Tabashnik 2000). The high fitness costs of target‐site and metabolic resistance were also qualitatively implicated through the rapid declines in RFs of laboratory‐evolved and field populations in the absence of pesticide selection (Figure 7B).

Narrow‐sense heritability was estimated to be very high at up to 0.387 over 10 generations (Table 2) leading to rapid resistance evolution (Figure 7A), but is expected to be orders of magnitude lower in the field because of fluctuating selection, higher mortality and greater environmental variance (Falconer 1960; McKenzie and Batterham 1998); though see (Weigensberg and Roff 1996). The experimental evolution never led to target‐site resistance, presumably because of the lower laboratory population sizes restricting the possibility of these mutations entering the population. Yet, the experimental evolution led to RFs as high as 741, which implies an unexpectedly high evolutionary potential for non‐target‐site resistance mechanisms.

### 4.4 Applications to Resistance Management

As well as a case study of evolution in action, chlorantraniliprole resistance in *C. suppressalis* and other lepidopteran pests in China also provides a cautionary tale for resistance management. When chlorantraniliprole arrived in the market in 2008, pesticides with other modes of action either had high levels of resistance or were banned. With such a devastating pest as *C. suppressalis*, it was unsurprising that chlorantraniliprole quickly and widely acquired intensive use—without reasonable scope for following IRAC recommendations for the rotation or mixture of modes of action (IRAC 2021, 2023). The repeated solo use of chlorantraniliprole led to the rapid evolution of resistance; the recommended use of a high dose is arguably likely to have delayed its onset, but otherwise no early measures were put in place to prevent what happened. This may seem surprising, but decisive action is constrained by market conditions and communication challenges, with users tending to prefer the most cost‐effective solution in the short term when choosing amongst comparable alternatives.

It was not predictable where the first reports of resistance (i.e., control failure) would come from, besides the major rice growing regions. This is only further emphasised in the years shortly after those first reports, where the level of resistance was spatially heterogeneous. Into this uncertainty, there was a great value to resistance monitoring, in identifying where resistance problems originate, are currently and will likely occur next. Resistance monitoring is expensive, but it enables informed intervention—even if that opportunity for a programme of resistance containment was not taken up in this case. Again, this may seem surprising, but coordinating a response is often a huge practical challenge.

After its establishment, the spread of resistance between locations becomes more predictable—not least in this case because the critical level of resistance leading to control failure was driven by the local spread of single major mutations (of the target site). Mathematical modelling can be used to develop strategies to delay the evolution and spread of resistance. Instead, in this case, resistance spread unchecked. From 2008 to the present, the case of chlorantraniliprole resistance evolution in *C. suppressalis* and other lepidopteran pests in China presents an illustrative example of how rapidly evolution can destroy the tools that are depended on to control pests.

## Conflicts of Interest

The authors declare no conflicts of interest.

## Data Availability

Data for this study are available at the Dryad Digital Repository: https://doi.org/10.5061/dryad.3ffbg79x2.
